# Cost-effectiveness analysis of malaria chemoprophylaxis for travellers to West-Africa

**DOI:** 10.1186/1471-2334-10-279

**Published:** 2010-09-22

**Authors:** Lukas L Widmer, Patricia R Blank, Koen Van Herck, Christoph Hatz, Patricia Schlagenhauf

**Affiliations:** 1 University of Zürich Centre for Travel Medicine, WHO Collaborating Centre for Travellers' Health, Hirschengraben 84, 8001 Zürich, Switzerland; 2 Institute of Social and Preventive Medicine, Departement of Health Economics, University of Zurich, Hirschengraben 84, 8001 Zurich, Switzerland; 3 Centre for the Evaluation of Vaccination, Vaccine & Infectious Disease Institute (VAXINFECTIO), University of Antwerp, Campus Drie Eiken (R2.17b), Universiteitsplein 1, B-2610 Antwerp, Belgium

## Abstract

**Background:**

The importation of malaria to non-endemic countries remains a major cause of travel-related morbidity and a leading cause of travel-related hospitalizations. Currently they are three priority medications for malaria prophylaxis to West Africa: mefloquine, atovaquone/proguanil and doxycycline. We investigate the cost effectiveness of a partial reimbursement of the cheapest effective malaria chemoprophylaxis (mefloquine) for travellers to high risk areas of malaria transmission compared with the current situation of no reimbursement.

**Methods:**

This study is a cost-effectiveness analysis based on malaria cases imported from West Africa to Switzerland from the perspective of the Swiss health system. We used a decision tree model and made a literature research on the components of travel related malaria. The main outcome measure was the cost effectiveness of malaria chemoprophylaxis reimbursement based on malaria and deaths averted.

**Results:**

Using a program where travellers would be reimbursed for 80% of the cost of the cheapest malaria chemoprophylaxis is dominant (i.e. cost saving and more effective than the current situation) using the assumption that currently 68.7% of travellers to West Africa use malaria chemoprophylaxis. If the current usage of malaria chemoprophylaxis would be higher, 82.4%, the incremental cost per malaria case averted is € 2'302. The incremental cost of malaria death averted is € 191'833.

The most important factors influencing the model were: the proportion of travellers using malaria chemoprophylaxis, the probability of contracting malaria without malaria chemoprophylaxis, the cost of the mefloquine regimen, the decrease in the number of travellers without malaria chemoprophylaxis in the reimbursement strategy.

**Conclusions:**

This study suggests that a reimbursement of 80% of the cost of the cheapest effective malaria chemoprophylaxis (mefloquine) for travellers from Switzerland to West Africa is highly effective in terms of malaria cases averted and is cost effective to the Swiss health system. These data are relevant to discussions about the cost effectiveness of malaria chemoprophylaxis reimbursement for vulnerable groups such as those visiting friends and relatives who have the highest risk of malaria, who are least likely to use chemoprophylaxis.

## Background

The importation of malaria to non-endemic countries remains a major cause of travel-related morbidity [1] and the leading cause of travel-related hospitalizations [2,3]. Over the last years the number of imported malaria decreased slightly in Switzerland and the UK but the proportion of *Plasmodium falciparum*, which is imported mainly from Sub-Saharan Africa and potentially fatal with a case fatality rate of 1.2% [1,4], increased [5,6].

Increased global mobility and ease of travel as well as the growing number of immigrants from malaria endemic countries have contributed to the importation of malaria in Europe. The immigrants from malaria endemic countries constitute a special risk group with high levels of malaria importation because often their status and financial circumstances hinder access to malaria chemoprophylaxis when they will visit their home country [7]. About half of the malaria patients treated in Switzerland have a foreign nationality [6]. West Africa is among the endemic regions with the highest estimated attack rates of malaria among travellers [5]. The Swiss Federal Office of Public Health (FOPH) reported an annual average of 112 malaria cases imported from West Africa for the years 2005 to 2008 [8]. This constitutes 60% of all reported malaria cases in Switzerland.

Malaria preventive measures include risk awareness, avoidance of mosquito bites and use of chemoprophylaxis. The current priority chemoprophylaxis regimens for Sub Saharan Africa are mefloquine, atovaquone/proguanil and doxycycline. As in many other Western countries, the Swiss public health system does not reimburse travellers for any of the recommended prophylactic agents. All of these anti-malaria medications have a similar prophylactic effectiveness although they vary in their adverse event profile and contraindications and also in cost [9]. The burden of the imported malaria cases in Switzerland is significant with an average of 307 imported cases and one to two deaths per year for the time period 1988 to 2002 [4]. Treatment and productivity losses are borne by the Swiss health system.

The aim of this study is to evaluate the cost-effectiveness of at least partial reimbursement of the cheapest drug for chemoprophylaxis compared with the current practice of no reimbursement for travellers medically insured in Switzerland, who visit West Africa. Partial reimbursement (80%) was chosen on the basis of prior analyses and the assumption that reimbursement will increase the use of (recourse to) malaria chemoprophylaxis.

## Methods

### Study design

This study is a cost-effectiveness analysis based on malaria cases imported from West Africa to Switzerland and was done from the perspective of the Swiss health system. Our work did not require ethics committee clearance because we used available published data on malaria risk and malaria associated costs. No human subjects were recruited for the analysis. West Africa was defined by OECD-definition as Benin, Burkina Faso, Cameroon, Chad, Ivory Cost, Ghana, Guinea, Guinea-Bissau, Gambia, Mali, Mauritania, Niger, Nigeria, Liberia, Senegal, Sierra Leone, Togo [10]. Cape Verde was excluded because it is not a malaria risk area.

**Figure 1:**
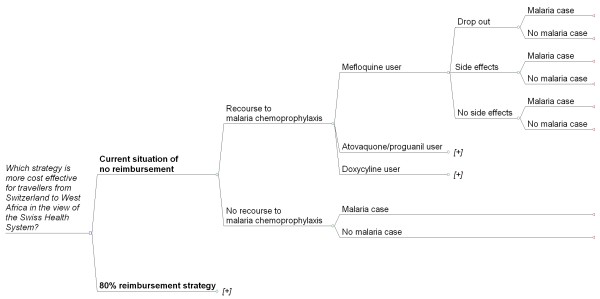
**Decision tree modelled to estimate cost and effectiveness of an 80% reimbursement of the cheapest malaria chemoprophylaxis regimen for travellers from Switzerland to West Africa compared to the current situation of no reimbursement**. The [+] signals the same tree as above in the figure.

A decision tree model (as shown in Figure 1) was developed using TreeAge Pro 2009 (TreeAge Software, Williamstown WA). We estimated the impact of an 80% reimbursement for the cheapest malaria chemoprophylaxis regimen or a voucher for the equivalent sum of money ("*80% reimbursement strategy" *label in tables). In the model we compared that strategy to the situation of no reimbursement, which is the current situation in Switzerland ("*current situation of no reimbursement*" label in tables). We calculated the cost for malaria chemoprophylaxis for a two week stay in West Africa (Table 1). So, in the proposed 80% reimbursement strategy every traveller who is prescribed malaria chemoprophylaxis would get a discount of € 17.77 (i.e. 80% of € 22.21 for mefloquine as Mephaquin^®^).

**Table 1:** The pattern of use of malaria chemoprophylaxis in Switzerland and the cost for the traveller of each option for a two week stay

Malaria chemoprophylaxis	Distribution of recourse to malaria chemoprophylaxis (based on records from the Swiss Tropical Institute, Basel)	Cost of malaria chemoprophylaxis for a two week stay in the current situation of no reimbursement	Cost of malaria chemoprophylaxis for a two week stay in the 80% reimbursement strategy
Mefloquine: Mephaquin^® ^8 tb box for € 22.21	55.4%	€ 22.21 (maximal protection 21 days)	€ 4.20

Atovaquone/proguanil: Malarone^®^ 12 tb box for € 41.88	40.7%	€ 83.75 (maximal 15 protection days)	€ 65.99

Doxycycline: Supracyclin^®^ 20 tb or 10 tb box for € 11.91 or € 6.40	3.9%	€ 30.23 (maximal protection 20 days)	€ 12.47

Travellers were divided in two theoretical groups: 1) those who didn't take any chemoprophylaxis and 2) those who used chemoprophylaxis. The chemoprophylaxis users were divided between the three recommended regimens mefloquine, atovaquone/proguanil and doxycycline. The decision tree takes adverse events into account. The traveller may experience adverse events (side effects) leading to withdrawal, or may experience adverse events without withdrawal or may not experience adverse events from the malaria chemoprophylaxis. At the end, the probability of contracting malaria is reduced (or not) by the prophylactic effectiveness of the chosen malaria chemoprophylaxis regimen. The probability of contracting malaria for withdrawals was estimated to be equivalent to those travellers who didn't use malaria chemoprophylaxis.

The 80% reimbursement strategy presents the same decision tree as the current situation of no reimbursement but differs on two points: the probability of recourse to malaria chemoprophylaxis and the respective cost of 80% of the price for mefloquine.

The value to compare the two situations was determined by calculating the cost and the health outcomes. The main outcome measure is given as incremental cost-effectiveness ratio (ICER) in terms of incremental cost per averted malaria case. Other outcome measures included the number of malaria cases, deaths and averted malaria cases for the reimbursement strategy, the average cost of malaria and associated chemoprophylaxis and the probability of contracting malaria per journey to West Africa.

### Input data

The estimates of variables used in the model were probabilities and medical costs stemming from a review of the literature and are summarized in Table 2.

**Table 2:** Estimates used to determine parameters for the decision tree model*

Definition of probabilities	Estimates (likliest, 95% CI)	Date of data	References
Current probability of recourse to malaria chemoprophylaxis in current situation of no reimbursement	0.687/0.55/0.824	2002-2003	[12-15]

Probability of recourse to malaria chemoprophylaxis in the 80% reimbursement strategy	0.865/0.806/0.925	-	[11,16]

Probability of recourse to mefloquine	0.554	2007	Personal evaluation at the STI^1^

Probability of recourse to atovaquone/proguanil	0.407	2007	Personal evaluation at the STI^1^

Probability of recourse to doxycycline	0.039	2007	Personal evaluation at the STI^1^

Probability of adverse events due to mefloquine leading to withdrawal	0.04 (0.01-0.08)	1998-2001	[9]

Probability of adverse events due to atovaquone/proguanil leading to withdrawal	0.02 (0.00-0.04)	1998-2001	[9]

Probability of adverse events due to doxycyline leading to withdrawal	0.03 (0.00-0.06)	1998-2001	[9]

Probability of severe adverse events due to mefloquine without withdrawal	0.11 (0.06-0.15)	1998-2001	[9]

Probability of severe adverse events due to atovaquone/proguanil without withdrawal	0.07 (0.02-0.11)	1998-2001	[9]

Probability of severe adverse events due to doxycyline without withdrawal	0.06 (0.02-0.10)	1998-2001	[9]

Probability of contracting malaria without malaria chemoprophylaxis	0.0242 (Varied ± 30%: 0.017 - 0.0314)	-	[18]

Prophylactic effectiveness of mefloquine	0.945 (0.84-0.981)	1993-1995	[19]

Prophylactic effectiveness of atovaquone/proguanil	0.958 (0.915-0.975)	Metaanalysis 2007	[20]

Prophylactic effectiveness of doxycyline	0.926 (0799-0.975)	1995	[21]

Rate of hospitalisation of the imported malaria cases in Switzerland	0.63	2003-2006	Personal evaluation with data from FOPH^2 ^and SFSO^3^

			

**Medical cost variables**	**Estimates (likliest)**	**Date of data**	**References**

Direct cost of mefloquine (Mephaquin^®^) for two weeks in 80% reimbursement strategy	€ 17.77	2008	[17]

Cost to treat adverse events	€ 37.59	2008	Personal evaluation with TARMED^4 ^and Swiss drug compendium[17]

Direct cost of hospitalisation due to a malaria case	€ 4763	2004-2005	Personal communication (SFSO)

Direct cost of ambulant treatment due to a malaria case	€ 368	2008	Personal communication (STI)

Average indirect cost of sick leave due to a malaria case	€ 2123	1996-2004	Personal communication (SUVA^5^)

*using the template created by Pistone[11]

^1 ^Swiss Tropical Institute

^2 ^Federal Office of Public Health (FOPH)

^3 ^Federal Statistical Office (SFSO)

^4 ^Swiss TARMED

^5 ^Swiss accident insurance funds

### Estimates on probabilities

#### Recourse to malaria chemoprophylaxis

Three different scenarios regarding the current recourse to malaria chemoprophylaxis for travellers to West Africa were analysed. We used 55% as the current probability of recourse to malaria chemoprophylaxis based on data from Pistone et al. [11] using studies of French residents [11-15]. The other two scenarios are conducted with an estimation of recourse to malaria chemoprophylaxis of 68.7% and a high estimation of 82.4% stemming from the data of the European airport study [12]. We estimated the increase in use of malaria chemoprophylaxis in the 80% reimbursement strategy to be 80.6%, 86.5%, and 92.5% using the methods of Pistone et al. for their 65% reimbursement strategy,. Their assumption stems from the result of the, 'enqûete santé protection sociale', ESPS (French healthcare and insurance survey), in 2004 [16]. Applied to our study, the introduction of the 80% reimbursement strategy would lead to an estimated 57% decrease in travellers from Switzerland to West Africa without a malaria chemoprophylaxis. Thus the calculation for the scenario of 68.7% usage of chemoprophylatic regimens, with 31.3% not taking malaria chemoprophylaxis would be as follows: 31.3% * (100% - 57%) = 13.5%. This means: the use of malaria chemoprophylaxis in the 80% reimbursement strategy would be 86.5% (100%-13.5%) compared to no reimbursement with 68.7% usage.

#### Distribution of recourse to the different recommended malaria chemoprophylaxis regimens

The distribution of use of the chemoprophylactic medications (Table 1) was evaluated from 644 travellers at the Swiss Tropical Institute (STI) in Basel. The recommended agents for West Africa are mefloquine, atovaquone/proguanil or doxycycline. For our calculations we used the least expensive drug in the Swiss drug compendium ie mefloquine as Mephaquin^® ^[17].

#### The probability of contracting malaria in West Africa without malaria chemoprophylaxis

We estimated the average length of stay for travellers to West Africa to be 14 days. Depending on this travel duration we estimate that the probability of contracting malaria in West Africa without malaria chemoprophylaxis would be 2.4% per two week of stay based on data from Steffen et al. [7,11,18].

#### The probabilities of severe adverse events and withdrawals

We used the study from Schlagenhauf et al. for the probabilities of severe adverse events and withdrawals [9]. Severe adverse events were defined as events requiring medical advice. Serious adverse events that require admission to hospital are extremely rare. The majority of the adverse events were of neuropsychological or gastrointestinal nature. We assumed that the probability of contracting malaria for the 'withdrawals' is the same as for those who do not take malaria chemoprophylaxis and for travellers with adverse events is the same as without adverse events.

#### The effectiveness of malaria chemoprophylactic regimens

The estimates of the prophylactic effectiveness are based on a review of literature on travel medicine malaria studies [19-21].

### Estimates on costs

The costs are given in Euro (€) and were converted Swiss Francs (CHF) to Euro (€) in 2009: 1 Swiss Franc (CHF) = 0.660 Euro (€) = 0.575 British Pound (£) = 0.936 US Dollar ($).

#### The cost for the malaria chemoprophylactic regimens

The figures on the cost for malaria chemoprophylaxis in Table 1 are from the official Swiss drug compendium 2008 [17]. The price for malaria chemoprophylaxis was calculated for an adult person staying a minimum of 14 days in West Africa.

#### The cost to treat severe adverse events

We estimated that a person with severe adverse events needed a ten minute consultation at the physician and a prescription for drug against nausea and vomiting plus one follow up visit of ten minutes. We used for our calculation the price for a package of Torecan^® ^(thiethylperazine). On the basis of the medical costs system in Switzerland, TARMED, and the 'Swiss compendium of drug information' we estimated the cost for treatment of severe adverse events at € 37.59.

#### The cost to treat a malaria case

The estimation of € 4'763 for the direct cost of a hospitalized malaria tropical case stems from unpublished data of the 'Swiss Federal Statistical Office' (SFSO), http://www.bfs.admin.ch/. The Swiss Tropical Institute estimated that an average ambulant malaria treatment including two physician consultations, hematology and malaria parasite laboratory confirmation plus a treatment course of Riamet^® ^(artemether/lumefantrine) to be € 368. The rate of hospitalized malaria patients was evaluated from reported cases in Swiss Federal Office of Public Health (FOPH) and the SFSO. Thus, the average treatment cost of a malaria case (based on both hospitalised and ambulatory cases) was calculated to be € 3'137. The average indirect cost of sick leave due to a malaria case (days of work loss) of hospitalized and ambulant treated patients, are calculated on the basis of unpublished data from the Swiss Accident Insurance Fund, SUVA, and are estimated to be € 2'123 [22].

### Analysis

The results are presented as incremental cost effectiveness ratio (ICER) in terms of cost per averted malaria case using an 80% reimbursement of the cheapest malaria chemoprophylaxis compared with the current situation where there is no reimbursement for malaria prophylaxis. ICER is calculated by dividing the incremental cost of the reimbursement strategy by the savings gained in the malaria cases. Using data from the Swiss Federal Statistical Office (SFSO) we estimated the annual number of Swiss travellers to West Africa to be 70'000 [23]. The estimation on the annual cost and effects (number of malaria cases) was conducted with the given number of travellers from Switzerland to West Africa. Costs and benefits were not discounted given the evaluation of malaria chemoprophylactic prevention and treatment would occur within a year.

To test for the precision and robustness of the model-results, deterministic sensitivity analyses were performed. For the one way sensitivity analysis, all model parameters were assigned a uniform probability distribution where possible, allowing ± 30% variability around the baseline. The variables on efficacy and probability of drop out, adverse events and no adverse events of malaria chemoprophylactic regimens were varied in their confidence interval. Furthermore, uncertainties around the result of the base-case were assessed in a probabilistic sensitivity analysis by using 10,000 sets of parameter values which are randomly sampled from a beta-distribution reflecting the ranges of variations given in Table 2[24].

## Results

### Cost and effect of the current situation and the 80% reimbursement strategy

The results are shown in Table 3 for the situation of recourse to malaria chemoprophylaxis of 55%, 68.7% and 82.4%. The absolute results calculated for 70'000 travellers to West Africa are presented in Table 4. The 80% reimbursement strategy leads in all situations to a significantly lower probability of contracting malaria per traveller and also to significantly lower costs per traveller in the situation of 55% and 68.7% recourse than in the current situation of no reimbursement. Only in the 82.4% recourse situation the cost would be higher in the 80% reimbursement strategy. Assuming current use of malaria chemoprophylaxis at 55% the reduction in malaria cases is 47% with an 22% decrease of cost (€ 65.45 to € 50.94per traveller); for a 68.7% recourse the reduction of malaria cases is 45% with a 9% reduction of cost (€ 50.05 to € 45.36per traveller); for a 82.4% recourse the reduction of malaria cases is 38% with 9% higher costs (€ 36.64 to € 39.78 per traveller).

**Table 3:** Relative cost and effects of the cost effectiveness analysis shown for the three different estimates on current recourse to malaria chemoprophylaxis

Cost-effectiviness analysis with the three different estimated 'recourse to malaria chemoprophylaxis'	55%		68.70%		82.40%	
	
	Current situation of no reimbursement	80% reimbursement strategy	Current situation of no reimbursement	80% reimbursement strategy	Current situation of no reimbursement	80% reimbursement strategy
Cost per journey (€) (95% CI)	65.45 (47.88 - 86.03)	50.94 (41.25 - 62.36)	50.05 (36.76 - 65.50)	45.36 (37.38 - 54.96)	36.64 (25.49 - 45.46)	39.78 (33.42 - 47.74)

Effect per journey (%) (95% CI) = probabilty of contracting malaria	0.01196 (0.00861 - 0.01587)	0.00625 (0.00445 -0.00836)	0.00891 (0.00640 - 0.01184)	0.00494 (0.00348 - 0.00669)	0.00586 (0.00417 - 0.00786)	0.00363 (0.00249 - 0.00506)

Cost Effectiveness ratio (€)						

Per case of malaria prevented	-	8'151	-	9'186	-	10'969

Per malaria related death prevented	-	679'250	-	765'490	-	914'045

Incremental cost (€) of 80% reimbursement strategy						

Per additional malaria case prevented	Reference strategy	dominant	Reference strategy	dominant	Reference strategy	2'302

Per additional malaria related death prevented	Reference strategy	dominant	Reference strategy	dominant	Reference strategy	191'833

**Table 4:** Absolute cost and effects calculated for an estimated 70'000 travellers from Switzerland to West Africa for the three different estimates on current recourse to malaria chemoprophylaxis

Cost-effectiviness analysis with the three different estimated 'recourse to malaria chemoprophylaxis'	55%		68.70%		82.40%	
	
	Current situation of no reimbursement	80% reimbursement strategy	Current situation of no reimbursement	80% reimbursement strategy	Current situation of no reimbursement	80% reimbursement strategy
Estimated number of travellers from Switzerland to West Africa	70'000					

Cost per year (€) likliest	4'581'654	3'565'716	3'503'346	3'175'326	2'425'038	2'784'936

Effect per year likliest						

Malaria cases	837	438	624	346	410	254

Malaria-related deaths	10	5	7	4	5	3

Effectiveness of 80% reimbursement strategy						

Malaria cases prevented	-	399	-	278	-	156

Malaria-related deaths prevented	-	5	-	3	-	2

In a scenario with 68.7% usage of chemoprophylaxis, the net cost for the public health system would be € 328'300 and a reduction of 278 malaria cases (from 624 to346), if the 80% reimbursement of the cheapest malaria chemoprophylaxis would be introduced (Table 4).

### Cost-effectiveness analysis of the 80% reimbursement strategy

The main medical effectiveness of the model is given in the number of malaria cases prevented and secondarily by the number of malaria related deaths prevented. The results are summarized in Table 3 and 4.

The 80% reimbursement strategy is dominant (i.e. cost saving and more effective than the current situation) in both situations of 55% and 68.7% recourse to malaria chemoprophylaxis. For the 82.4% recourse situation the incremental cost per additional malaria case prevented is € 2'302 and for prevention of one malaria related death € 191'833.

These results underline the high influence of the current situation on the recourse to malaria chemoprophylaxis in our model.

### The sensitivity analysis

The one-way sensitivity analysis on the cost of the 80% reimbursement strategy shows the highest sensitivity for the following variables: probability of recourse to malaria chemoprophylaxis, probability of contracting malaria without malaria chemoprophylaxis, cost of mefloquine regimen, the decrease in the number of travellers without malaria chemoprophylaxis in the reimbursement strategy. All other variables have a minor effect on the incremental cost effectiveness ratio. The effects on the incremental cost effectiveness ratio per averted malaria case are presented in Table 5 for these parameters and the ICER of reimbursing atovaquone/proguanil or doxycycline instead of mefloquine are shown.

**Table 5:** One way sensitivity analysis on the incremental cost effectiveness ratio (ICER) for selected input factors with the highest influence on the model shown for a current usage on malaria chemoprophylaxis of 68.7%

Scenario	€/'malaria cases averted'*
Current probability of recourse to malaria chemoprophylaxis	

Very low (40%)	dominant

Low (55%)	dominant

High (82.4%)	2'302

Probability of contracting malaria without malaria chemoprophylaxis for a two week stay	

Extremly low (= 0.4%)	19'899

Very low (= 0.9%)	5'922

Low, (= 1.2%)	3'126

A bit low, -30% (= 1.7%)	548

High, +30% (= 3.1%)	dominant

Cost of the mefloquine drug	

Low, -30% (= € 15.54)	dominant

High, +30% (= € 28.86)	dominant

Decrease of 'non-malaria chemoprophylaxis-users' due to the 80% reimbursement	

Low, -30% (40%)	126

High, +30% (74%)	dominant

Reimbursement of the more expansive drugs	

Doxycycline (€ 30.23)	dominant

80% reimbursement of all three drugs	4'968

Percentage of reimbursement of the cheapest malaria chemoprophylaxis	

Very low (60%)	dominant

Low (70%)	dominant

High (90%)	dominant

Very High (100%)	dominant

*the incremental cost effectiveness ratio (ICER) per averted malaria case

**Figure 2:**
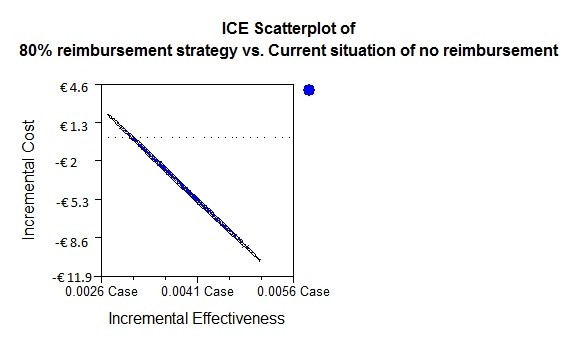
**Incremental Cost Effectiveness (ICE) scatterplot: probabilistic sensitivity analysis shown for the situation of 68.7% recourse to malaria chemoprophylaxis**.

The probabilistic sensitivity analysis (Figure 2) resulted in a 95% probability of being dominant in the situation of 68.7% recourse to malaria chemoprophylaxis based on data from Table 2.

## Discussion

The cost-effectiveness analysis in this study suggests that an 80% reimbursement of the cheapest chemoprophylaxis regimen in Swiss travellers to West Africa could lead to a substantial reduction in malaria cases and be cost effective to the Swiss Health System. The evaluation of the three scenarios on recourse to malaria chemoprophylaxis showed that the results are highly dependent on the input variable 'use of or recourse to malaria chemoprophylaxis' for which only inaccurate data exist. A low use of chemoprophylaxis as seen in immigrant travellers could make this strategy highly interesting for the decision makers.

### Strength and weaknesses

This study gives a good overview about imported malaria from a high risk area in the form of a model with the all important variables in it. We tried to include as many direct factors as possible in the model while also keeping it as simple as possible. It has to be remembered that we conducted a cost effectiveness analysis with the comparison between the current situation of no reimbursement and a strategy of 80% reimbursement of the cheapest malaria chemoprophylaxis. The model is robust and changes in input data such as underestimations of cost of treating malaria cases or hospitalization rate will not unduly influence the outcome. The model can also easily be adapted to more precise input factors or to other population group with a different situation on imported malaria.

A weakness of the analysis is the lack of exact data on the input factor 'use of or recourse to malaria chemoprophylaxis' and this factor has a major impact on the ICER (incremental cost effectiveness ratio). There were no specific data on chemoprophylaxis use available for Swiss residents. So we used an estimation based on European residents derived from the data of the airport study which was conducted in nine different airports in Europe with passengers residing in Europe and boarding a flight to West Africa [12]. This information is, however, not representative, because targeted destinations and flights were pre-selected for practical reasons, the study participation was on voluntarily basis and unprepared travellers were probably less likely to participate. This meant a bias in favour of those using chemoprophylaxis and a resulting overestimation of recourse to malaria chemoprophylaxis. The three studies from France, which Pistone et al. analyzed for travellers to West Africa, might underestimate the recourse to malaria chemoprophylaxis for Swiss travellers [13-15]. The proportion of immigrants from West Africa living in France is higher than in Switzerland. Several studies have shown that visiting friends and relatives (VFR) are at increased risk of acquiring malaria [5,7]. One of the reasons is the lower recourse to malaria chemoprophylaxis.

The incidence of malaria in travellers, who stopped taking malaria chemoprophylaxis (withdrawals), was assumed to be the same as travellers without malaria chemoprophylaxis. But they could still have a limited protection from the taken malaria chemoprophylaxis and it would overestimate the incidence of malaria.

In our model we considered only the three recommended chemoprophylactic agents for West Africa from the Swiss Federal Office of Public Health (FOPH): mefloquine, atovaquone/proguanil and doxycyline. Others, like chloroquine, which has a partial prophylactic effectiveness, were excluded from the model. This could lead to an overestimation of the incidence of malaria cases.

The increase in malaria chemoprophylaxis users for our 80% reimbursement strategy was assumed based on the study of Pistone et al. [11]. We made a conservative assumption and took the same factor (57%) for the increase of malaria chemoprophylaxis-users as Pistone et al. in their 65% reimbursement strategy. This would possibly underestimate the ICER. But it has to be remembered that travellers seeking travel advice in a health clinic still have to pay for the visit by themselves.

The number of travellers to West Africa was estimated using air transportation statistics, but it included only travellers with a flight ticket where the final destination was an airport in West Africa. Travellers flying to a destination in West Africa via secondary routes are not registered. This fact has no influence on the incremental cost and effectiveness as well as the ICER, but they have a main impact on the absolute number of malaria cases, malaria related deaths and their associated cost for the Swiss health system. This makes the validation of our model more difficult. The results of the model with the estimated annual number of travellers of 70'000 overestimated in all three situation of recourse to malaria chemoprophylaxis the number of malaria cases reported at the Swiss Federal Office of Public Health (FOPH). The origin of the malaria acquisition is not known for every reported malaria case and may underestimate the true number of malaria cases from West Africa. Furthermore, the model does not include malaria cases that are treated outside of Switzerland.

The 80% reimbursement strategy of only the cheapest malaria chemoprophylaxis (mefloquine) makes the proportional prices of the three recommended malaria chemoprophylaxis agents very diverse: for a two week stay the price reduction for mefloquine would be 80%, for doxycyline 59% and for atovaquone/proguanil 21%. These percentages change with chosen brand names and travel duration. In Switzerland, broken packs are not dispensed in this situation. This idea of giving a voucher for malaria chemoprophylactic medication equivalent to 80% of the cost of the cheapest anti-malaria regimen is a new and an innovative approach to increase the recourse to prophylactic drugs. Mefloquine has the advantage of wide applicability (including small children, pregnant women, long-term travellers) and is comparable with other malaria chemoprophylaxis in its prophylactic effectiveness and frequency of severe adverse events but is associated with a high incidence of non-serious adverse events, particularly in women, that may impact on well being [9]. Contraindications for mefloquine are: active depression, a recent history of depression, generalized anxiety disorder, psychosis, or schizophrenia or other major psychiatric disorders, or with a history of convulsions. The similar prophylactic efficacy of the three regimens, differing mainly in their contraindications, makes this 80% reimbursement of the cheapest malaria chemoprophylactic agents a reasonable proposition. The realization of the 80% reimbursement strategy of the cheapest malaria chemoprophylaxis, mefloquine, for a journey to West Africa could be made by delivering a voucher for the given travel duration approved by a valid individual ticket for a destination in West Africa.

### Comparison with other studies

In the literature, one other cost-effectiveness analysis has been conducted from the perspective of the French health system for travellers to Sub-Saharan Africa and the results showed that the incremental cost of their 65% reimbursement strategy has a positive cost-effectiveness impact [11]. Two older studies analyzed cost minimization in use of malaria chemoprophylaxis in travellers and concluded that malaria chemoprophylaxis is cost-effective from the perspective from the British health care system and also from the German and Swiss systems respectively [25,26].

### Meaning of the study

In most economic analyses, where two strategies or more are compared, the incremental cost effectiveness ratio, ICER, is given in life years gained or QALY (quality adjusted life years). This study used averted malaria cases as ICER and not QALYs. The National Institute for Health and Clinical Excellence (NICE), is a specific health authority of the National Health Service (NHS) in Britain which gives guidelines for appropriate treatment regimens for different diseases. NICE considers a treatment intervention with a threshold of € 22'962 to € 34'443 (£20'00 to £30'000) per QALY to be cost effective. If our study results were expressed in terms of QALYs, the theoretical calculations are as follow:

The average age at death due to malaria in Switzerland is 51.3 years [4]. The life expectancy in Switzerland is around 83 years [27]. That would lead to 31.7 life years lost. If we assumed that the average quality of these 31.7 life years is 0.8 it would be 25 QALY. The costs of the 80% reimbursement for one malaria related death averted is € 7673 (€ 191'833 divided by 25 QALY), using the study study assumption that 82.4% of travellers to West Africa use chemoprophylaxis, The 80% reimbursement is considered cost effective according to the NICE guidelines.

### Implications for research and policy

This study attempts to provide economic data on the costs of malaria prevention and the expenses associated with imported malaria in industrialized countries. This analysis is based on traveller data. Travellers to West Africa are not homogeneous but comprise tourist, business travellers and increasing numbers of "visiting friends and relative" travellers. Currently in Switzerland the main burden of malaria is borne by this last group and future research can possibility focus on this group in economic evaluations as some studies have shown that chemoprophylaxis use in this group falls far short of the 55% recourse to chemoprophylaxis assumed in this analysis [7]. Other countries with large migrant populations can also consider such economic evaluations for malaria and travel-related illness.

## Conclusion

An 80% reimbursement of the cheapest malaria chemoprophylaxis or a voucher of the equivalent amount of money for an alternative chemoprophylactic regimen for travellers from Switzerland to West Africa is expected to be highly effective in terms of malaria cases averted and is cost-effective for the Swiss health system. In certain groups of high risk travellers, such as VFR travellers, chemoprophylaxis use is much lower and here an 80% reimbursement would be highly cost effective. These data are relevant to discussions about the cost effectiveness of malaria chemoprophylaxis reimbursement for vulnerable groups such as those visting friends and relatives.

## Competing interests

None of the authors have any conflict of interest with the contents of this paper. Dr. Schlagenhauf has received research funding, consulting fees and/or speakers' honoraria from Hoffmann-La Roche, Glaxo Smith Kline and Pfizer.

## Contributions

All authors have seen and approved the final version of the paper. LW was involved in the concept, design, analysis and writing/revising the paper. He is the guarantor. PB helped with the concept and revision of the paper and gave major input in the analysis. KvH contributed data and critically revised the paper. CH contributed data. PS was involved in the concept, design, analysis and writing/revising the paper and she is the project supervisor. All authors read an approved the final draft.

## Pre-publication history

The pre-publication history for this paper can be accessed here:

http://www.biomedcentral.com/1471-2334/10/279/prepub
